# Tattoos, piercings, and symptoms of ADHD in non-clinical adults: a cross-sectional study

**DOI:** 10.3389/fpsyt.2023.1224811

**Published:** 2024-01-03

**Authors:** Martin Ragnar Glans, Joel Nilsson, Susanne Bejerot

**Affiliations:** School of Medical Sciences, Faculty of Medicine and Health, Örebro University, Örebro, Sweden

**Keywords:** impulsivity, hyperactivity, inattention, tattoo, piercing

## Abstract

**Introduction:**

Tattoos and piercings are associated with impulsive and risk-taking personality traits, which are also common along the ADHD continuum. However, studies on ADHD and body modification are lacking. Thus, this study aimed to assess the association between body modification and subclinical ADHD symptom severity and to investigate if body modification can serve as an indication for ADHD examination.

**Methods:**

A total of 762 adults (529 women and 233 men) without a diagnosis of ADHD completed the adult ADHD Self-Report Scale (ASRS) and answered questions concerning body modification. Two different ASRS versions were utilized: the 18-item ASRS Symptom Checklist and the 6-item ASRS Screener. Three categorizations of body modifications were analyzed: (i) having at least one tattoo, (ii) having at least one piercing other than ear piercing, and (iii) the combination of simultaneously having at least one tattoo and one piercing. Mean 18-item ASRS total and subscale scores and the proportion of positive results on the 6-item ASRS Screener were compared between those with and those without body modifications while adjusting for covariates age and sex. Additional analyses were performed for ≥2 and ≥3 body modifications.

**Results:**

In our cohort, 26% had a tattoo, 14% had a piercing other than ear piercing, and 8% had a combination of tattoo and piercing. Having any kind of body modification was associated with more pronounced symptoms of ADHD and with a cutoff score on the ASRS screener indicating ADHD. Whereas, the effect sizes were small for tattoos, medium to large effect sizes were seen for ≥2 piercings in the ASRS. Moreover, moderately strong associations emerged for ≥1 piercing and a positive ASRS screening result.

**Conclusion:**

Our results suggest that acquiring a body modification, especially a tattoo, is entering the mainstream in Sweden. Correspondingly, differences in subclinical ADHD symptomatology between non-clinical adults with and without body modifications are subtle. Having ≥2 piercings other than ear piercings, on the other hand, is associated with clinically relevant differences in ADHD symptoms. Moreover, piercing status may serve as an indicator, among others, for further ADHD assessments. However, more research is needed to ascertain the possible signaling functions of body modifications in clinical settings.

## Introduction

Tattooing and piercing have been a part of human culture for thousands of years and have held different significance depending on the culture and era. Up until the 20th century, tattoos in Western societies were mainly associated with certain groups, such as soldiers, sailors, bikers, and criminals (1, 2). At the beginning of the 21st century, body modifications were met by negative attitudes. A survey from 1998 found that healthcare staff demonstrated negative biases against tattooed people (3). Moreover, individuals with visible tattoos and/or piercings risked facing discrimination in the job market (4–7). However, over the past decades, previously held stigmas appear to have attenuated (8–10). Body modifications have become more common in Western societies, although reported prevalence rates vary considerably depending on country, demographics, and time of the study. For tattoos, they typically range ~10–20% (8), and for piercings, ~10–50% (9, 11, 12).

From a psychiatric viewpoint, it has been suggested that tattoos and piercings may serve as an indirect marker for personality traits (13, 14). Tattoos and/or piercings have been associated with characteristics such as impulsiveness, “sensation seeking,” “need for uniqueness,” and being less agreeable (e.g., friendly/compassionate) and less conscientious (e.g., careful, diligent, and thorough) (15–17). Moreover, tattooed individuals express impaired inhibitory control, elevated impulsiveness, and risky decision-making compared to non-tattooed individuals (18, 19). Tattoos and piercings are also associated with impulse-related behaviors such as alcohol consumption, violence, illicit drug use, and suicide (20–23). Additionally, forensic studies on autopsy records have reported an association between tattoos and early mortality (24), whereas findings on the relationship between tattoos and homicide are somewhat conflicting (25, 26). Finally, tattoos and/or piercings have been linked with borderline personality disorder (BPD) (27) and antisocial personality disorder (28), both of which are highly associated with ADHD (29). Yet, also here, findings have been ambiguous, and one Polish study found no difference between adults with and without tattoos and/or piercings in terms of psychopathology, as assessed by self-report (30).

Thus, based on previous research and clinical experience, an association between body modification and ADHD could be expected. However, to the best of our knowledge, no previous studies have investigated this potential association. Moreover, following the shift from a categorical to a dimensional conceptualization of ADHD (31, 32), we believe that there is a motivation to examine potential associations across the full ADHD continuum. Therefore, the aims of the present study were the following: (i) to evaluate the relationship between modern-day body modifications and subclinical ADHD symptom severity among non-clinical adults, (ii) to investigate if body modification may serve as a clinical marker for further ADHD examination, and (iii) to assess if the number of acquired body modifications impacts the putative associations.

## Method

### Participants and procedure

A convenience sample was recruited between 2014 and 2019 from various settings, including healthcare professionals attending a mandatory mental health course (*n* = 414), students on university campus (*n* = 238), patients and next-of-kin recruited in a health center waiting room (*n* = 112), and staff at various workplaces (*n* = 82). Participation involved anonymously completing a questionnaire including a measure of ADHD symptoms, questions about body modification, and demographic data. Inclusion criteria included speaking Swedish, being between the ages of 18 and 65 years, and not having been diagnosed with ADHD. Exclusion criteria included any missing data on ADHD diagnosis, tattoo and piercing status, age, or sex. For the 18-item Adult ADHD Self-Report Scale (ASRS), we allowed one missing item from each subscale. In such cases, we used the mean substitution method. For the six-item ASRS Screener, no missing data were allowed.

The study was approved by the medical ethical review board in Stockholm, Dnr. 2014/1742-31 and Dnr 2017/2140-32. Written informed consent was obtained from all subjects.

### Measures

Symptoms of ADHD were assessed by two variants of the WHO Adult ADHD Self-Report Scale (ASRS-v1.1): the 18-item ASRS Symptom Checklist and the 6-item ASRS Screener. Whereas, the 18-item version is an inventory of symptom burden, the 6-item Screener was developed to screen for individuals at risk for adult ADHD.

The 18-item ASRS (33) measures the presence and frequency of current symptoms of ADHD. It consists of 18 items reflecting the DSM-IV symptoms/criteria for ADHD, divided into two 9-item subscales on hyperactivity/impulsivity (ASRS Hy/Imp) vs. inattention (ASRS Inatt). Consistent with the idea of ADHD traits as one dimension and to retain information about trait values, a continuous scoring method of 0–4 (0 = never, 1 = rarely, 2 = sometimes, 3 = often, and 4 = very often) was used for the 18-item ASRS, yielding a total range of 0–72, with each subscale ranging from 0 to 36. A significant correlation (*r* = 0.43) between total scores and clinical symptom severity has been demonstrated, supporting the use of the continuous scoring method (33). In a Norwegian study of a non-psychiatric population, the mean ASRS total score was 23.5 in men and 22.2 in women (34).

The 6-item ASRS Screener is a subset of the 18-item ASRS and comprises two items on hyperactivity/impulsivity and four items on inattention. When screening for ADHD, the recommendation is to sum dichotomized responses across all items, yielding a total range of 0–6. A cutoff score of ≥4 indicates possible ADHD. To transform the five different grades for each item into a dichotomous scale of 0 (absence) or 1 (presence), cut points for “clinically significant symptom levels” were determined. The cutoff varies between different items; for items 1–3, this was set as “sometimes”, and for items 4–6, this was set as “often”. The six-item ASRS Screener was reported to have a sensitivity of 68.7% and a specificity of 99.5% (33). In a large population-based study from Sweden, 6.8% of the participants scored positive on the ASRS Screener (35).

### Body modification

Tattoo and piercing status was self-reported based on two questions: “How many tattoos do you have?” and “How many piercings other than ear piercings do you have?” The full piercing question is hereafter referred to as “piercing” unless explanations are needed for clarity. Ear piercings were not included because they were considered culturally appropriate, consistent with previous studies (15, 21). For the analyses, we categorized body modification status into three grouping variables: tattoo status, piercing status, and the combination of having at least one tattoo and piercing (s) simultaneously. Each grouping variable was dichotomized as (i) at least one tattoo vs. no tattoo, (ii) at least one piercing vs. no piercing, and (iii) the combination of having at least one tattoo and piercing simultaneously vs. no combined tattoo and piercing. Additional analyses on tattoo and piercing status were performed for the cutoffs ≥2 and ≥3 tattoos and piercings, respectively.

### Statistics

Two variants of the ASRS were evaluated: the 18-item ASRS Symptom Checklist Scale and the 6-item ASRS Screener. First, the mean 18-item ASRS total and subscale scores were compared between those with and without body modification by Student's *t*-tests and Cohen's *d* effect size. Cohen's *d* effect size was considered small for values ≤ 0.2, medium for ≈0.5, and large for ≥0.8. The assumption of normality for the ASRS scores was assessed by the visual inspection of Q–Q plots. To further control the effects of deviation from the normal distribution and outliers, sensitivity analyses by supplementary Mann–Whitney *U*-tests were performed. Since comparisons of mean values on the ASRS were preferred, we chose to only present Student's *t*-test if both methods produced statistically significant results. Second, the proportion of positive results on the six-item ASRS Screener was compared between those with and without body modification using chi-square tests. Adjusted analyses for potential covariates age (in years) and sex (male/female) were performed by the use of multiple linear and logistic regression models. Dependent variables were the 18-item ASRS total score, ASRS Hy/imp subscale score, ASRS Inatt subscale score, and the 6-item ASRS Screener (yes/no). The independent variable of interest was body modification status (i.e., tattoo, piercing, and tattoo-piercing combined). The *P*-values reported are two-sided. All statistical analyses were performed in SPSS version 28.

## Results

**Figure 1:**
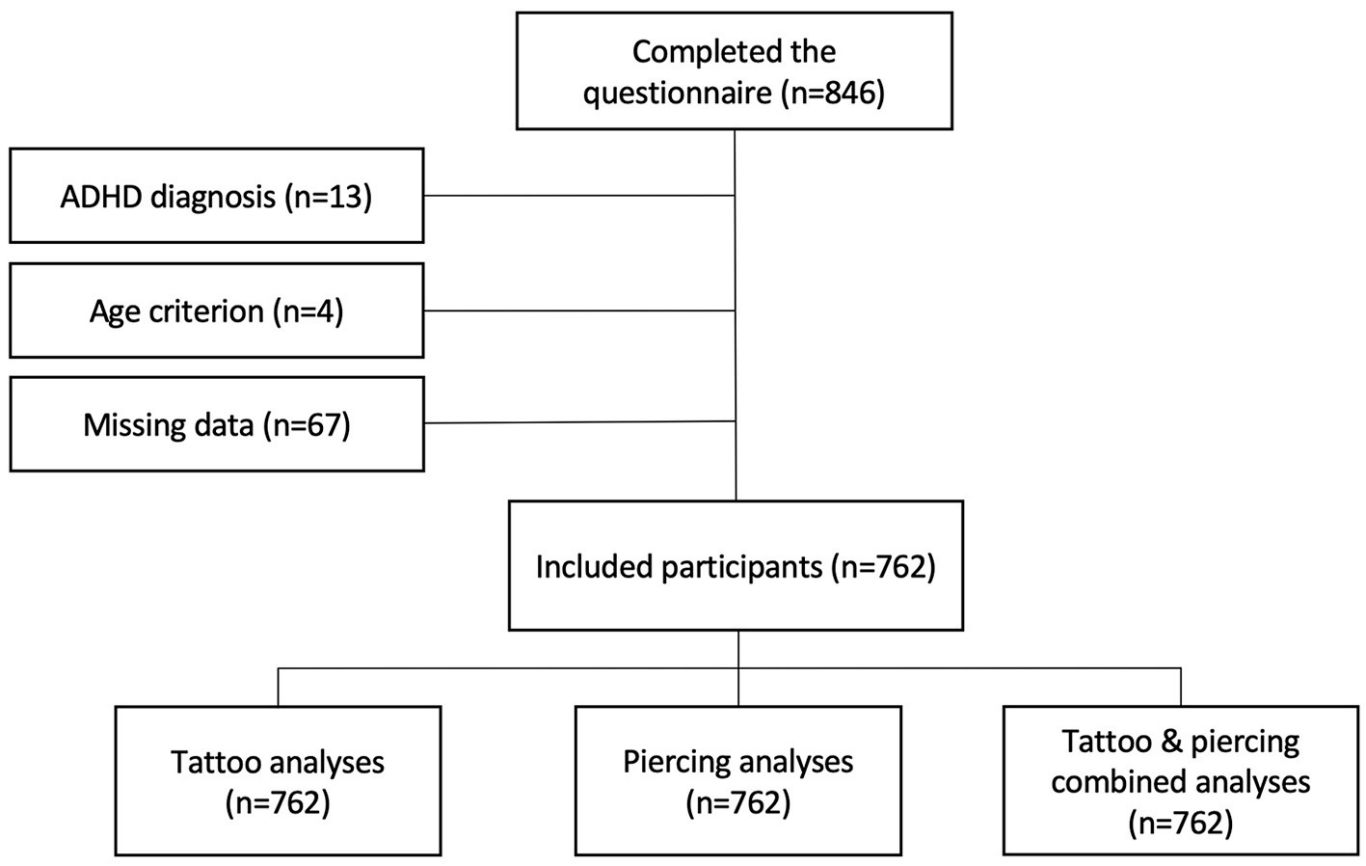
Flowchart of study participants.

A total of 762 participants (529 women and 233 men) met the overall inclusion and exclusion criteria for the study (Figure 1). An additional 21 participants (nine women and 12 men) were excluded from the six-item ASRS Screener analyses. In our cohort, 26% had a tattoo, 14% had a piercing other than ear piercing, and 8% had a combination of tattoo and piercing. Mean (SD) ASRS scores for the full sample were 27.7 (9.3) and 27.9 (10.0) for the ASRS total score, 13.2 (5.4) and 13.1 (5.8) for the hyperactivity/impulsivity subscale, and 14.5 (5.1) and 14.8 (5.3) for the inattention subscale, for women and men, respectively. The assumption of approximate normality for the ASRS scores was satisfied for all group combinations of body modification, as assessed by the visual inspection of Q–Q plots. The proportion of positive results on the six-item ASRS screener in the full sample was 16.7% for women and 22.2% for men. The characteristics of the study population and body modification status stratified on sex are presented in Table 1. Demographic variables for the different body modification statuses are presented in Supplementary Table 1.

**Table 1:** Characteristics of the study population, *N* = 762.

Female sex, *n* (%)	529 (69.4)
**Age (yrs), mean, (SD)**	
Women	38.1 (13.3)
Men	34.9 (12.9)
**Employment status, *n* (%)**	
Working	412 (54.1)
Student	230 (30.2)
Unemployed	3 (0.3)
Missing data	117 (15.4)
**Body modification status, *n* (%)**	
**≥1 tattoos, 199 (26.1)**	
Women	166 (31.4)
Men	33 (14.2)
**≥2 tattoos, 94 (12.3)**	
Women	79 (14.9)
Men	15 (6.4)
**≥3 tattoos, 57 (7.5)**	
Women	47 (8.9)
Men	10 (4.3)
**≥1 piercing other than ear piercing, 106 (13.9)**	
Women	98 (18.5)
Men	8 (3.4)
**≥2 piercings other than ear piercing, 58 (7.6)**	
Women	56 (10.6)
Men	2 (0.9)
**≥3 piercings other than ear piercing, 30 (3.9)**	
Women	29 (5.5)
Men	1 (0.4)
**≥1 tattoo and ≥1 piercing combined, 59 (7.7)**	
Women	57 (10.8)
Men	2 (0.9)

*n*, number; SD, standard deviation; yrs, years.

### The 18-item ASRS symptom checklist

Having any kind of body modification was associated with higher scores on the ASRS total scale and hyperactivity/impulsivity subscale, whereas statistically significant differences between groups in the inattention subscale only emerged for the piercing analyses. The differences in ASRS scores were larger for piercing and piercing and tattoo combined compared to tattoo-only status. Moreover, an increased number of piercings, but not tattoos, resulted in more pronounced differences in the ASRS scores (Table 2). Comparisons between groups, including crude and adjusted differences in the ASRS scores, are presented in Table 2. A detailed presentation of the linear regression models, adjusting for the potential covariates age and sex, is given in Supplementary Table 2.

**Table 2:** Comparisons of ASRS total and subscale scores between participants with and without any body modification.

	**Body modification**			**Difference in ASRS score**	
	**Yes**	**No**		**Crude**	**Adjusted**
**ASRS scores**			***d***	**Difference**, ***p*****-value**	
**Tattoo status**					
**ASRS total score, mean (SD)**					
≥1 tattoos vs. no tattoos	29.4 (9.5)	27.2 (9.4)	0.23	2.2, **0.005**	2.0, **0.010**
≥2 tattoos vs. < 2 tattoos	29.2 (9.6)	27.6 (9.5)	0.17	1.6, 0.120	1.2, 0.232
≥3 tattoos vs. < 3 tattoos	28.9 (9.7)	27.7 (9.5)	0.13	1.2, 0.355	0.9, 0.486
**ASRS hyperactivity/impulsivity subscale, mean (SD)**					
≥1 tattoos vs. no tattoos	14.2 (5.6)	12.8 (5.5)	0.25	1.4, **0.003**	1.3, **0.007**
≥2 tattoos vs. < 2 tattoos	14.4 (5.6)	13.0 (5.5)	0.24	1.3, **0.028**	1.1, 0.068
≥3 tattoos vs. < 3 tattoos	14.4 (5.9)	13.1 (5.5)	0.24	1.3, 0.086	1.1, 0.138
**ASRS inattention subscale, mean (SD)**					
≥1 tattoos vs. no tattoos	15.2 (5.3)	14.4 (5.1)	0.16	0.82, 0.055	0.77, 0.074
≥2 tattoos vs. < 2 tattoos	14.8 (5.5)	14.5 (5.2)	0.06	0.29, 0.617	0.131, 0.819
≥3 tattoos vs. < 3 tattoos	14.5 (5.2)	14.6 (5.2)	−0.02	−0.10, 0.887	−0.224, 0.753
**Piercing status**					
**ASRS total score, mean (SD)**					
≥1 piercings vs. no piercings	31.0 (9.9)	27.2 (9.3)	0.40	3.8, ** < 0.001**	3.3, **0.001**
≥2 piercings vs. < 2 piercings	32.1 (9.1)	27.4 (9.4)	0.49	4.7, ** < 0.001**	4.5, ** < 0.001**
≥3 piercings vs. < 3 piercings	33.8 (9.6)	27.5 (9.4)	0.67	6.3, ** < 0.001**	6.1, ** < 0.001**
**ASRS hyperactivity/impulsivity subscale, mean (SD)**					
≥1 piercings vs. no piercings	15.3 (5.9)	12.9 (5.4)	0.44	2.4, ** < 0.001**	2.1, ** < 0.001**
≥2 piercings vs. < 2 piercings	15.3 (5.7)	13.0 (5.5)	0.42	2.3, **0.002**	2.2, **0.004**
≥3 piercings vs. < 3 piercings	16.6 (5.9)	13.1 (5.5)	0.64	3.5, **0.001**	3.3, **0.001**
**ASRS inattention subscale, mean (SD)**					
≥1 piercings vs. no piercings	15.7 (5.3)	14.4 (5.2)	0.26	1.4, **0.012**	1.2, **0.033**
≥2 piercings vs. < 2 piercings	16.7 (5.0)	14.4 (5.2)	0.45	2.3, **0.001**	2.3, **0.001**
≥3 piercings vs. < 3 piercings	17.2 (5.0)	14.5 (5.2)	0.54	2.8, **0.004**	2.7, **0.005**
**Tattoo and piercing combined**					
**ASRS total score, mean (SD)**					
≥1 tattoo and ≥1 piercings	31.5 (10.0)	27.5 (9.4)	0.43	4.1, **0.001**	3.3, **0.010**
**ASRS hyperactivity/impulsivity subscale, mean (SD)**					
≥1 tattoo and ≥1 piercings	15.5 (6.1)	13.0 (5.5)	0.46	2.5, ** < 0.001**	2.1, **0.006**
**ASRS inattention subscale, mean (SD)**					
≥1 tattoo and ≥1 piercings	16.0 (5.3)	14.4 (5.2)	0.30	1.6, **0.027**	1.2, 0.081

SD, standard deviation; Dif, difference; *d*, Cohen's *d*; *p, p*-value.

Body modification status was self-reported. First, bivariate analyses compared mean ASRS scores between those with and without body modification using Student's *t*-test. Cohen's d describes effect sizes for the bivariate analyses. Second, multiple linear regression assessed the impact of body modification status (yes/no) on the ASRS scores while adjusting for age (years) and sex (male/female). Adjusted difference refers to unstandardized B, i.e., the degree of change in the outcome variable (ASRS score) for every 1 unit of change in the predictor variable of interest (body modification/no body modification). All *p*-values are two-sided. Bold values denote statistical significance at the *p* < 0.05 level.

### The six-item ASRS Screener

Having any body modification was associated with a higher proportion of individuals screening positive results on the six-item ASRS Screener; 24 vs. 17%, *p* = 0.028 for tattoos, 32 vs. 16%, *p* > 0.001 for piercing, and 33 vs. 17%, *p* = 0.002 for tattoo and piercing combined. Comparisons between groups, as well as crude and adjusted odds ratios for a positive result on the six-item ASRS Screener, are presented in Table 3. A detailed presentation of the logistic regression models, adjusting for potential covariates such as age and sex, is given in Supplementary Table 3.

**Table 3:** Proportion of positive results on the six-item ASRS Screener compared between participants with and without body modification.

	**Positive ASRS, *n* (%)**	**Risk estimate**					
		**Crude**			**Adjusted**		
		**OR**	**(95% CI)**	***P***	**OR**	**(95% CI)**	***P***
**Tattoo status**							
≥**1 tattoo vs. no tattoo**							
Yes	45 (23.7)	1.6	(1.0–2.3)	**0.028**	1.7	(1.1–2.6)	**0.013**
No	91 (16.5)						
≥**2 tattoos vs**. < **2 tattoos**							
Yes	22 (24.2)	1.5	(0.89–2.5)	0.126	1.5	(0.91–2.6)	0.109
No	114 (17.5)						
≥**3 tattoos vs**. < **3 tattoos**							
Yes	12 (21.8)	1.3	(0.65–2.5)	0.490	1.3	(0.62–2.6)	0.446
No	124 (18.1)						
**Piercing status**							
≥**1 piercing vs. no piercings**							
Yes	33 (31.7)	2.4	(1.5–3.8)	**< 0.001**	2.6	(1.6–4.3)	**< 0.001**
No	103 (16.2)						
≥**2 piercings vs**. < **2 piercings**							
Yes	18 (32.1)	2.3	(1.3–4.1)	**0.006**	2.6	(1.4–4.7)	**0.003**
No	118 (17.2)						
≥**3 piercings vs**. < **3 piercings**							
Yes	12 (41.4)	3.3	(1.6–7.2)	**0.001**	3.7	(1.7–8.1)	**0.001**
No	124 (17.4)						
**Tattoo and piercing combined status**							
≥**1 Tattoo and** ≥**1 piercing**							
Yes	19 (33.3)	2.4	(1.3–4.4)	**0.002**	2.5	(1.4–4.7)	**0.003**
No	117 (17.1)						

*n*, number; OR, odds ratio; *P, p*-value.

Body modification status was self-reported. First, bivariate analyses compared the proportion of positive results on the six-item ASRS Screener within participants with and without body modification using Pearson's chi-square test. Fisher's exact test was used when the expected value of a cell was < 5. Second, multivariate analyses for the association between body modification status (yes/no) and a positive result on the six-item ASRS Screener, while adjusting for age (years) and sex (male/female) were performed using multiple logistic models. All *p*-values are two-sided. Bold values denote statistical significance at the *p* < 0.05 level.

## Discussion

In this study, we examined differences in symptoms of hyperactivity, impulsivity, and inattention between non-clinical adults with and without body modifications (i.e., tattooed, pierced, and the combination of having a tattoo and a piercing). To the best of our knowledge, this is the first study to evaluate these associations. In accordance with our hypothesis, body modifications were associated with more pronounced subclinical ADHD symptoms. Whereas, the effect sizes for tattoo status were rather small, medium to large effect sizes were found for piercing. Moreover, an increasing number of piercings, but not tattoos, resulted in larger differences in the ASRS scores between groups. Thus, a greater deviation from the mainstream appears to correlate with a more prominent subclinical ADHD phenotype. Finally, a moderately strong association was found for having a piercing other than ear piercing and scoring above the cutoff on the six-item ASRS Screener, indicating that piercings, among others, may serve as a clinical marker when assessing ADHD. It should be emphasized that we assessed a non-clinical cohort in the present study. This approach followed the conceptualization of a dimensional, rather than a qualitatively distinct nature of ADHD characteristics.

### ADHD symptom severity

The first aim of the study was to evaluate the association between body modification and subclinical ADHD symptom severity. Statistically significant results, albeit with weak effect sizes, emerged when we compared differences in the 18-item ASRS scores between body-modified and non-modified subjects. Unadjusted differences in ASRS total scores were 2.2 for ≥1 tattoo and 3.8 for ≥1 piercing as compared to individuals without such body modifications. The effect sizes were 0.23 and 0.40, respectively. These findings are consistent with contemporary research on body modification and personality profiles and suggest that the strength of the association between body modification and psychopathology is smaller than previously reported or that it has attenuated over time (17, 36). The prevalence rates of body modification in our cohort, particularly having a tattoo, suggest that it is entering the cultural mainstream in Sweden, aligning with the trend toward normality observed in other studies (8, 9). In our cohort, 31% of the women and 14% of the men reported having at least one tattoo. Piercing, on the other hand, was less common; 19% of women and 3% of men reported having at least one piercing other than an ear piercing, and there was a considerable drop in prevalence for more than two piercings.

Whereas, the effect sizes for tattoo status were rather small, medium to large effect sizes were found for piercing. Likely, the more mainstream a phenomenon becomes, the more heterogeneous and less distinguished the group of individuals partaking in the phenomenon will be. Accordingly, increasing the cutoff for the number of piercings but not for tattoos resulted in clinically significant differences between groups with regard to the ASRS scores. The crude differences in mean ASRS total scores were 4.7 points for ≥2 vs. < 2 piercings and 6.3 points for ≥3 vs. < 3 piercings, which corresponded to effect sizes of 0.5 and 0.7, respectively. Moreover, the piercing variable and the tattoo and piercing combined variable revealed similar effect sizes. Thus, the qualitatively decisive factor appears to be linked with acquiring a piercing and not a tattoo.

Naturally, a number of things influence whether someone decides to acquire a tattoo and/or a piercing. Whether a slightly more pronounced ADHD phenotype plays an important role in decision-making remains speculative and unlikely to be decisive. Various incentives for getting tattooed, e.g., attaining a beauty accessory, enhancing one's individuality, or as an expression of values, have been reported (1). In our study cohort, the hyperactivity/impulsivity subscale revealed larger effect sizes than the inattention subscale. It is reasonable to believe that many of the aforementioned motivations are related to impulsivity rather than inattention. Decisions about having a tattoo or piercing may be related to both impulsiveness and sensation seeking. These characteristics are associated with testing limits, in contrast to what can presumably be associated with inattentiveness.

Given tattoos' lasting nature compared to piercings, our finding that piercings had a larger effect size is somewhat surprising. This challenges the idea that impulsivity is a primary driver for pursuing body modifications. However, it is important to note that our analyses did not consider qualitative aspects of the body modification apart from excluding ear piercings. Consequently, the piercing variable may have selected individuals with culturally inappropriate body modifications to a greater extent than the tattoo variable.

### Body modification as an indication of ADHD

The second aim of the study was to evaluate the association between body modifications and signs of ADHD. For this purpose, we applied the six-item ASRS Screener, which is a widely used screening tool for ADHD. Moderately strong associations emerged for the piercing variables and a positive screening result. For tattoos, the adjusted OR was 1.7 (95% CI: 1.1–2.6), for piercings 2.6 (95% CI: 1.6–4.3), and for combined tattoo and piercing 2.5 (95% CI: 1.4–4.7). This indicates that information on piercings and tattoos may add value to the ADHD assessments. Given today's liberal attitude concerning body modifications, the patient need not necessarily feel uncomfortable with such questions during the clinical interview.

Noteworthy, the six-item ASRS Screener focuses on symptoms of inattention, with four items addressing inattention and two items on hyperactivity. In our analyses of the ASRS total and subscale scores, the most pronounced differences were found for the hyperactivity/impulsivity subscale. Whether the six-item ASRS Screener measures two separate constructs or is largely unidimensional remains unsettled (35, 37–39).

The current study focused on the subclinical part of the ADHD spectrum. Expanding on these findings, future research should include both clinical and non-clinical cohorts and evaluate the predictive value of body modifications for a definitive ADHD diagnosis. Moreover, prevalence comparisons of tattoos and piercings between individuals with and without ADHD would be enabled. Finally, comparing clinical and non-clinical cases with similar ASRS scores could help clarify whether the presumed association is more influenced by underlying symptoms or the identity linked to an ADHD diagnosis.

## Study strengths and limitations

The current study has a number of strengths. First, validated and commonly distributed instruments to assess ADHD symptoms were used. Second, the large study sample enabled multiple analyses and the ability to adjust for covariates that could potentially confound the association. Third, participants were recruited from various places, which presumably improved the reliability of our findings.

The results of the present study also have to be interpreted in light of its limitations. All data in this study were self-reported, and no methods of verification ensured the correctness of the responses. However, because we did not ask private questions such as the reasons for body modification or which body part was chosen, and because participation was anonymous, accurate responses could be expected. Moreover, the relatively high reported prevalence rates of body modification and results on ASRS show no indications of censored reporting.

The use of a convenient sampling method affects the generalizability of the results. The vast majority of participants were seemingly high-functioning and in employment or pursuing studies. Including a greater socio-demographic spread would have increased the generalizability. Moreover, the low number of men (*n* = 8) reporting having a piercing clearly limits the generalizability of our results for men. Nevertheless, the significant results seen in the primary analyses remained in the adjusted analyses, which support our overall conclusions. We lack information on exact response rates, but we estimate drop-out to be below 10% as we (SB and MG) collected all data on site from the participants.

We did not obtain information on the location, size, or appearance of the body modification, which prevented analyses regarding how conventional the body modification was. It is likely that there are qualitative differences with regard to factors such as how much a body modification deviates from the norm, what it portrays, or to which subgroup it may be affiliated. Moreover, we excluded all ear piercings. Probably, it would have been more correct to only deem soft earlobe piercings as culturally appropriate.

We did not collect data concerning smoking, alcohol use and illicit drug use, factors known to be associated with body modifications as well as ADHD, and consequently with higher ASRS scores.

The cross-sectional design prevents conclusions about the temporal aspect of the association. Possibly, obtaining a tattoo or piercing affects an individual's self-image and how they will respond in self-reports such as the ASRS. In other words, the act of getting a body modification might cause self-perceived differences between body-modified and non-modified individuals. Furthermore, the signaling functions of tattoos and piercings may vary depending on the cultural and geographical setting in which the body modification was acquired, which was not addressed in the present study. Finally, we did not inquire when in life a tattoo or piercing was acquired. An older individual would have had a longer time to acquire a body modification as opposed to a younger individual, who might still get one later in life. During what time period a person was young might also have influenced their propensity to get a body modification, since the acceptance of acquiring a body modification has changed over the years. However, statistically significant results remained after adjusting the analyses for age, which suggests that age was not influential on the overall results in this study.

We did not obtain information regarding the presence of co-existing symptoms of BPD or other psychiatric conditions. ADHD and BPD share important clinical features such as emotional dysregulation and impulsivity (40, 41), but low self-esteem and impaired interpersonal relationships are frequent in both disorders (42). In a recent study on prevalence rates of body modifications among individuals with BPD, subjects with ADHD were used as a control group. The study revealed that individuals with BPD had a significantly higher prevalence of body modifications compared to those with ADHD (43). Furthermore, they found a significant association between emotional dysregulation and the total number of piercings in BPD patients, leading the authors to suggest that body modification might serve as a coping mechanism for emotion regulation. The present study design did not enable adjusted analyses with regard to BPD. Nonetheless, we believe that our results motivate further exploration of the relationship between body modification and ADHD phenotypes. However, for a comprehensive understanding of psychopathological factors involved in body modification, future studies should examine qualitative features from both the ADHD and BPD symptom dimensions. Equally, for a comprehensive evaluation of the potential differential-diagnostic qualities of tattoos and piercings, studies should ideally incorporate additional psychiatric cohorts (e.g., bipolar disorder, Tourette syndrome, and other types of personality disorders).

## Conclusion

The results of the present study suggest that differences in subclinical ADHD symptoms between body-modified and non-modified individuals from the general population are subtle, in particular with regard to tattoos or just having one piercing other than an ear piercing. In addition, previously held assumptions about tattooed individuals need to be re-evaluated as having a tattoo is entering the mainstream in Western societies. Having ≥2 piercings other than ear piercing, on the other hand, was associated with more pronounced ADHD symptoms, with moderately large effect sizes. Thus, a greater deviation from the mainstream appears to correlate with clinically relevant differences in subclinical ADHD symptomatology.

Moreover, our results indicate that body piercings may serve as a clinical indicator, among others, when deciding if further examination for ADHD is warranted. However, more research is needed to ascertain the possible signaling functions of body modifications in clinical settings.

## Data availability statement

The raw data supporting the conclusions of this article will be made available by the authors, without undue reservation.

## Ethics statement

The studies involving humans were approved by the Medical Ethical Review Board in Stockholm, Dnr. 2014/1742-31 and Dnr 2017/2140-32. The studies were conducted in accordance with the local legislation and institutional requirements. The participants provided their written informed consent to participate in this study.

## Author contributions

MG, JN, and SB were responsible for study conceptualization and design. MG was responsible for data collection. MG and JN conducted statistical analyses and drafted the manuscript. SB provided critical revision of the manuscript for important intellectual content. All authors approved the final version.
