# Supplementary material for: Tattoos, piercings, and symptoms of ADHD in non-clinical adults: a cross-sectional study
Source: Front Psychiatry. 2024 Jan 3;14:1224811. doi: 10.3389/fpsyt.2023.1224811 (PMC10791871; doi:10.3389/fpsyt.2023.1224811)
Supplement: Supplementary file 1 [file Table_1.pdf]

Supplementary Table 1. Demographics for the different body modification variables

Characteristic	Body modification status Yes	No	Test of difference
Tattoo analyses			
Sex female, n	166	363	
male	33	200	
Age (yrs), (mean, SD)	36.0 (11.1)	37.5 (13.9)	t = 1.3, p=.183
Employment status, n (%)			
Employed	133 (66.8)	279 (49.6)	χ^2 = 23.5, **p <.001**
Student	34 (17.1)	196 (34.8)	
Unemployed	1 (.5)	2 (.4)	
Missing data	31 (15.6)	86 (15.3)	
Piercing analyses			
Sex, female, n	98	431	
male	8	225	
Age (yrs), (mean, SD)	33.4 (11.1)	37.7 (13.5)	t = 3.1, **p = .002**
Employment status, n (%)			χ^2 = 4.5, p=.209
Employed	67 (63.2)	345 (52.6)	
Student	25 (23.6)	205 (31.3)	
Unemployed	0 (0.0)	3 (.5)	
Missing data	14 (13.2)	103 (15.7)	
Tattoo & piercing combined			
Sex, female, n	57	472	
male, n	2	231	
Age (yrs), (mean, SD)	31.6 (9.1)	37.6 (13.4)	t = 3.3, **p<.001**
Employment status, n (%)			χ^2 = 6.4, p=.094
Employed	41 (69.5)	371 (52.5)	
Student	11 (18.6)	219 (31.2)	
Unemployed	0 (0.0)	3 (.4)	
Missing data	7 (11.9)	110 (15.6)	

Abbreviations: n, number; p, p-value; SD, standard deviation; yrs, years

Note: Body modification status was self-reported. Continuous variables were analysed by the Student's *t*-test, and categorical variables were analysed by the Chi-squared test. All *p* values are 2-sided. Bold values denote statistical significance at the p < 0.05 level.
