# Supplementary material for: Tattoos, piercings, and symptoms of ADHD in non-clinical adults: a cross-sectional study
Source: Front Psychiatry. 2024 Jan 3;14:1224811. doi: 10.3389/fpsyt.2023.1224811 (PMC10791871; doi:10.3389/fpsyt.2023.1224811)
Supplement: Supplementary file 2 [file Table_2.pdf]

Supplementary Table 2. The results of multiple linear regression analysis for the impact of body modification on ASRS scores.

Predictors	Unstandardized B	SE	Standardized B	t	P
Tattoo status					
ASRS total score					
Body modification	2.020	.781	.094	2.59	**.010**
Sex	.079	.748	.004	.105	.916
Age	-.135	.026	-.188	-5.26	**<.001**
ASRS hyperactivity/impulsivity subscale					
Tattoo	1.250	.458	.099	2.73	**.007**
Sex	-.144	.439	-.012	-.328	.743
Age	-.071	.015	-.169	-4.70	**<.001**
ASRS inattention subscale					
Tattoo	.770	.430	.065	1.79	.074
Sex	.223	.412	.020	.541	.589
Age	-.064	.014	-.164	-4.54	**<.001**
Piercing status					
ASRS total score					
Body modification	3.30	.999	.120	3.30	**.001**
Sex	.251	.750	.012	.334	.738
Age	-.128	.026	-.178	-4.96	**<.001**
ASRS hyperactivity/impulsivity subscale					
Body modification	2.12	.586	.132	3.612	**<.001**
Sex	-.025	.440	-.002	-.057	.955
Age	-.066	.015	-.158	-4.37	**<.001**
ASRS inattention subscale					
Body modification	1.18	.552	.079	2.135	**.033**
Sex	.276	.414	.024	.666	.506
Age	-.062	.014	-.157	-4.343	**<.001**
Tattoo & piercing combined					
ASRS total score					
Body modification	3.34	1.29	.094	2.585	**.010**
Sex	.078	.748	.004	.104	.917
Age	-.130	.026	-.181	-5.029	**<.001**
ASRS hyperactivity/impulsivity subscale					
Body modification	2.09	.757	.101	2.76	**.006**
Sex	-.142	.439	-.012	-.323	.747
Age	-.068	.015	-.161	-4.46	**<.001**
ASRS inattention subscale					
Body modification	1.245	.711	.064	1.75	.081
Sex	.219	.412	.019	.532	.595
Age	-.062	.014	-.159	-4.380	**<.001**

Abbreviations: ASRS, Adult ADHD self-report scale; B, Beta; P, p-value; SE, Standard error

Note: Body modification status was self-reported. Multiple linear regression assessed the impact of body modification status (yes/no) on the ASRS scores, while adjusting for age (years) and sex (male/female). Unstandardized B reflects the degree of change in the outcome variable (ASRS score) for every 1-unit of change in the predictor variable of interest (body

modification/no body modification). All *p* values are 2-sided. Bold values denote statistical significance at the $p < 0.05$ level.
