# Supplementary material for: Tattoos, piercings, and symptoms of ADHD in non-clinical adults: a cross-sectional study
Source: Front Psychiatry. 2024 Jan 3;14:1224811. doi: 10.3389/fpsyt.2023.1224811 (PMC10791871; doi:10.3389/fpsyt.2023.1224811)
Supplement: Supplementary file 3 [file Table_3.pdf]

**Supplementary Table 3.** The results of multiple logistic regression analysis on the association between body modification status and a positive result on the 6-item ASRS Screener.

| Predictor | B | SE | Wald | *df* | p | Adjusted OR (95% CI) |
|---|---|---|---|---|---|---|
| Tattoo status | | | | | | |
| Body modification | .534 | .214 | 6.22 | 1 | **.013** | 1.7 (1.1-2.6) |
| Sex | .405 | .210 | 3.74 | 1 | .053 | 1.5 (.99-2.3) |
| Age | -.19 | .008 | 5.90 | 1 | **.015** | .98 (.97-.996) |
| Piercing status | | | | | | |
| Body modification | .968 | .250 | 15.0 | 1 | **<.001** | 2.6 (1.6-4.3) |
| Sex | .484 | .213 | 5.16 | 1 | **.023** | 1.6 (1.1-2.5) |
| Age | -.016 | .008 | 4.12 | 1 | **.042** | .98 (.97-.99) |
| Tattoo & piercing combined | | | | | | |
| Body modification | .934 | .312 | 8.99 | 1 | **.003** | 2.5 (1.4-4.7) |
| Sex | .415 | .210 | 3.93 | 1 | **.047** | 1.5 (1.0-2.3) |
| Age | -.016 | .008 | 4.51 | 1 | **.034** | .98 (.97-.99) |

*Abbreviations:* ASRS, Adult ADHD self-report scale; B, Beta; CI, confidence interval; OR, odds ratio; P, p-value; SE, Standard error.

*Note:* Body modification status was self-reported. Multiple logistic regression assessed the association between body modification status (yes/no) and a positive result on the 6-item ASRS Screener, while adjusting for age (years) and sex (male/female). All *p* values are 2-sided. Bold values denote statistical significance at the $p < 0.05$ level.
